# A cautionary note on the association between meteorological parameters and COVID-19 pandemic

**DOI:** 10.7189/jogh.10.020355

**Published:** 2020-12

**Authors:** Shola Adeyemi, Usame Yakutcan, Adekunle O Adeoti, Eren Demir

**Affiliations:** 1 Bohemian Smartlytics Ltd & Statsxperts Consulting Limited, Haverhill, UK; 2 University of Hertfordshire, Hertfordshire Business School, Hatfield, UK; 3 Faculty of Clinical Sciences, Department of Medicine, Ekiti State University, Ado-Ekiti, Ekiti State, Nigeria

Will the increasing temperature and humidity stop the spread of coronavirus, like seasonal patterns seen in viruses like influenza? In the authors’ opinion, weather has little or no part to play in bringing an end to the pandemic. As soon as the World Health Organization (WHO) declared the COVID-19 a pandemic, many published articles reported temperature and humidity as potential weather parameter that could wane off the daily confirmed COVID-19 cases [1,2].

COVID-19 pandemic has set the globe on a medical emergency by constituting a threat to human existence. A holistic and non-medically related approach to the reduction in disease burden is urgently required. Most countries have gradually tightened lockdown policies and citizens are recommended to stay at home and preserve the physical distance. On the other hand, this concept demands critical review of the meteorological parameters and its relationship with the disease transmission, morbidity and mortality of COVID-19 which has been a subject of research since its outbreak. Several postulations to the uneven disease burden in various regions were adduced to the climatic variations.

In Spanish province, there was no consistent evidence to suggest the relationship between the exponential COVID-19 cases and temperature values [3]. This finding is similar to the Chinese study on the association between mean temperature and transmission of SARS-CoV-2 in 122 cities of China where there was no evidence that warmer weather reduced the number of reported cases of COVID-19 [4]. However, studies relating extreme temperatures to the survival of other respiratory viral illnesses like SARS and influenza show that warmer temperature could reduce the spread of COVID-19. Sajadi et al [5] in study on global community transmission of COVID-19 suggested that the distribution along latitude, temperature, and humidity is similar to the pattern seen in the other seasonal respiratory virus. However, these meteorological parameters could conflict with the evolving body of knowledge which has ascribed the reduction in morbidity and mortality in COVID-19 to sunlight exposure and the effect of vitamin D [6].

The multi-indicator Oxford COVID-19 Government Response Tracker (OxCGRT) was designed to collect data on government policies such as school closures and travel restrictions in over 160 countries. These data constitute the stringency index (SI), which reflect the strictness of government policies but not necessarily the effectiveness of a country’s response [7]. Countries with high SI like India, where early implementation of containment policies were enforced reported slow progression in mortality and morbidity of COVID-19 [8].

Similarly, lessons on containment from previous coronavirus outbreaks like SARS and MERS were deployed in Singapore, Taiwan and South Korea which yielded good outcome. The reduction in carbon emission and its possible effect during containment and other climatic indicators like wind speed, humidity, and temperature have been explored as the major predictors in the spread of respiratory-related infectious diseases [9]. The critical role of climatic change was demonstrated in the New York city experience, a densely populated city in the USA, where an exponential increase in the number of confirmed cases significantly correlated with average temperature, minimum temperature, and air quality [10].

As concluded in Adeyemi et al [11], there are significant caveats in the literature, as related to statistical assumptions and the spatial-temporal nature of the data. Most of the publications reported temperature and humidity as potential factors that could wane off the daily confirmed COVID-19 cases. However, the correlation is not causal, thus it is difficult to validate these findings.

Adeyemi et al [11] reported an interesting finding in the correlations (70%-80%) between SI and the COVID-19 outcomes. The strongest was between SI and confirmed cases, meaning that the more confirmed cases, unsurprisingly the stricter the lockdown. We deduce that this is due to governments’ intensification of control measures in response to the surge in COVID-19 cases. This suggested that only effective containment strategies like lockdown stringency and not weather can substantially limit the spread of the pandemic. Similarly, causality or interdependencies were ruled out between any of the weather parameters considered in their research, and COVID-19 outcomes.

## FACTORS THAT IMPACT ON COVID-19 OUTBREAK: STRINGENCY TRUMPS WEATHER

As of late May 2020, the total number of confirmed COVID-19 cases surpassed 5 million worldwide, whereas no clear evidence of human-to-human transmission was reported in mid-January 2020 [12]. As the figures and fear in people escalated very quickly, the measures by the countries against the pandemic have varied a lot, from agile to none. Even, the virus is classified as a normal flu, or is denied as cannot be seen by human eye, or the word “coronavirus” is banned by some countries` leaders. In addition to the measures, the weather is seen an indicator reducing the spread. We show in this section why most of the association reported needs to be interpreted with caution and why they potentially might be spurious correlations.

**Figure 1:**
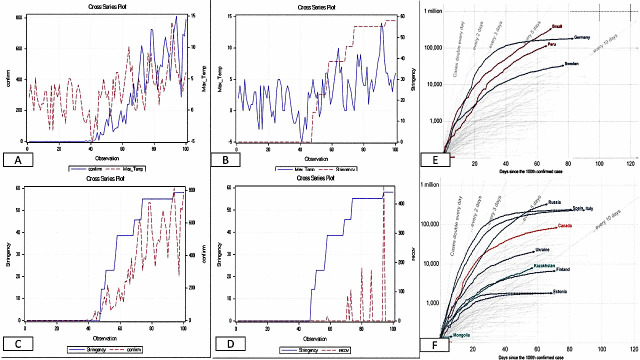
Cross series between variables. Panel A. Cross series of maximum daily temperature and confirmed cases for Sweden. Panel B. Cross-series of maximum temperature and stringency index for Sweden. Panel C. Cross-series of stringency index and recovered case for Sweden. Panel D. Cross series of stringency index and confirmed cases for Sweden. **Source**: Data retrieved for 22 January to 30th April 2020 from Dong et al. [12]. Panel E. Cumulative confirmed cases for Germany, Brazil, Peru, and Sweden. Panel F. Cumulative confirmed cases for Russia, Canada, Ukraine, Kazakhstan, Finland, Estonia, Iceland, Mongolia, Spain and Italy. Data retrieved for 22 January to30 April 2020 [13]. Stringency index data retrieved from Hale et al [7].

Generally, despite the welcoming of warm weather across Europe, countries are reporting more cases as testing is scaled up. Most of the countries with currently less cases of COVID-19 are those with early detection of the virus, caught up with testing with lockdown restrictions into place. While those who currently see more cases are those who put low restrictions into place, had the virus later and started doing more testing or those who relaxed their restrictions and had a resurgence of the virus. An example is Sweden, where restrictions were not immediately heightened, see **Figure 1**, panels A, B, C and D.

**Figure:**
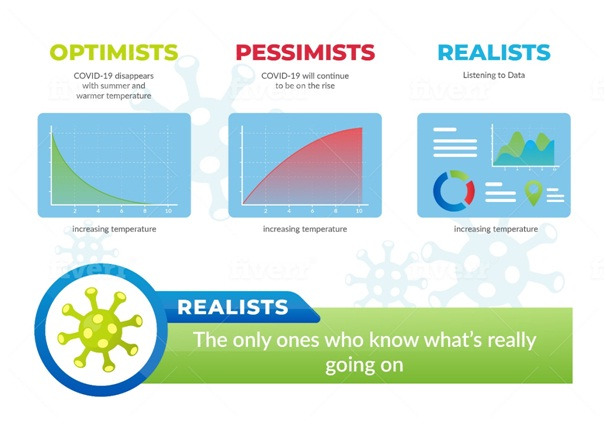
Photo: From the authors’ own collection, used with permission.

A look at Panel A in **Figure 1** would reveal that the number of confirmed cases is associated with high temperature, the cross-series plot reveals an upward trend. Panel B also reveals an upward trend in daily maximum temperature and SI. This is because as the temperature becomes favourable, people in Sweden tended to spend time outside. Based on this the government decided to implement a staged lockdown restriction. Looking at the Panel B more closely, you would see the lag effect of the dynamic between SI and maximum temperature. This is because the effect of SI kicks in a few days after implementation. Panel D reveals that as many more cases are being reported, as more stringency is being implemented. Panel C reveals the influence of the SI on recovery rate albeit the lag effect is more pronounced, which may be due to the haphazard reporting of recovery rates.

Panels E and F have presented cumulative cases across some of the warmest (E) and coldest (F) countries of the world. The cumulative reported cases for 4 different countries, namely Germany, Brazil, Peru, and Sweden, are presented in Panel E. The daily cases in Germany have started decreasing as we enter the spring and welcoming warm weather. However, this will be a spurious assumption that the warmer weather is causing the few daily cases being reported. The main driver is the stringency put in place by the German government which stood at 80 (SI) as at April 1, 2020. If warmer weather were to play any significant role in daily confirmed cases, then countries like Brazil and Peru should have never reached the current numbers. Despite the SI of 96 in Peru (starting April 1, 2020), the number of confirmed cases was on the rise, as Peruvians did not adhere to lockdown rules. Therefore, human behaviour was making the impact of the SI to be negligible. Brazil delayed its lockdown response for too long, and now paying a heavy price (as of mid-May 2020), with increasing number of deaths, and fears are growing that Brazil (and Latin America) could become the pandemic’s next epicentre [14]. Sweden has a different approach of herd immunity, with minor restrictions put in place, a SI of 58, and despite increasing temperature, there has been steady daily reported cases of COVID-19.

## SUMMARY AND CONCLUSION

The planet is facing the biggest threat against humankind in this century by a novel kind of wild virus. The countries and individuals, that underestimated the virus and the disease, are now paying a heavy and mournful price. People hope that the climate (eg, hot weather, humidity) might be one of the major factors stopping the spread. However, amongst the scientific community, there is no clear consensus on how meteorological parameters will impact COVID-19. Adeyemi et al [11] found serious limitations in the statistical assumptions made in these studies, which prompted them to exhaustively analyse data from 10 countries across 6 continents, examining the link between COVID-19 outcomes and meteorology parameters and other factors of interest, such as the stringency index. The key message is that statistical measures must be interpreted cautiously.

Therefore, in the absence of any vaccine and pharmacological therapy, stringency, human behaviours, and health care preparedness are the most effective ways of preventing the spread of virus, especially to minimise death in the elderly population and those with underlying medical conditions.
